# The Role of Astroglia in the Epileptic Brain

**DOI:** 10.3389/fphar.2012.00132

**Published:** 2012-07-12

**Authors:** Gabriele Losi, Mario Cammarota, Giorgio Carmignoto

**Affiliations:** ^1^Institute of Neuroscience of the National Research Council and Department of Biomedical Sciences, University of PadovaPadova, Italy

**Keywords:** astrocyte, epilepsy, seizures, glial-neuronal interactions, ictal event

## Abstract

Epilepsies comprise a family of multifactorial neurological disorders that affect at least 50 million people worldwide. Despite a long history of neurobiological and clinical studies the mechanisms that lead the brain network to a hyperexcitable state and to the intense, massive neuronal discharges reflecting a seizure episode are only partially defined. Most epilepsies of genetic origin are related to mutations in ionic channels that cause neuronal hyperexcitability. However, idiopathic epilepsies of unclear origin represent the majority of these brain disorders. A large body of evidence suggests that in the epileptic brain neurons are not the only players. Indeed, the glial cell astrocyte is known to be morphologically and functionally altered in different types of epilepsy. Although it is unclear whether these astrocyte dysfunctions can have a causative role in epileptogenesis, the hypothesis that astrocytes contribute to epileptiform activities recently received a considerable experimental support. Notably, currently used antiepileptic drugs, that act mainly on neuronal ion channels, are ineffective in a large group of patients. Clarifying astrocyte functions in the epileptic brain tissue could unveil astrocytes as novel therapeutic targets. In this review we present first a short overview on the role of astrocytes in the epileptic brain starting from the “historical” observations on their fundamental modulation of brain homeostasis, such as the control of water content, ionic equilibrium, and neurotransmitters concentrations. We then focus our review on most recent studies that hint at a distinct contribution of these cells in the generation of focal epileptiform activities.

## Introduction

Epilepsies are caused by genetic or acquired factors, such as brain injury, post-infection lesions, and tumors and affect at least 50 million people worldwide (Ngugi et al., 2010; Thurman et al., 2011). An epileptic seizure is the clinical manifestation of an abnormal and excessive discharge that initially involves neurons from a restricted brain site (the epileptogenic focus) and then propagates to nearby neuronal populations eventually spreading to the entire brain. Most currently used antiepileptic drugs (AEDs) target either voltage-sensitive ion channels or GABAergic signaling reducing neuronal excitability or increasing inhibition in the brain network, respectively (Rogawski and Loscher, 2004). These drugs are, however, ineffective in a large portion of epileptic patients and their anticonvulsant action is often accompanied by relevant side effects. Novel therapeutic targets are, therefore, highly demanded.

A different relatively unexploited strategy would be to target the glia cell astrocytes. These cells are key players in the control of the brain tissue homeostasis by regulating water content and K^+^ extracellular levels and by providing neurons with nutrients and trophic factors. Astrocytes play a prominent role in neurovascular coupling mechanism (Zonta et al., 2003; Takano et al., 2006; for recent reviews, see Carmignoto and Gomez-Gonzalo, 2010; Petzold and Murthy, 2011) and are also largely responsible for maintaining physiological levels of glutamate (Rothstein et al., 1994, 1996; Huang et al., 2004) and GABA in the extracellular space (Conti et al., 2004; Madsen et al., 2010). In addition, astrocytes participate with microglial cells in the defensive response against endo and exogenous insults by releasing various cytokines and pro-inflammatory mediators that ultimately have a proconvulsant action (Turrin and Rivest, 2004; Vezzani and Granata, 2005; Vezzani et al., 2008; Fabene et al., 2010).

While the astrocytic contribution to these processes is well recognized since many decades, more recent works have greatly extended the role of astrocytes from the control of brain tissue homeostasis to the modulation of synaptic transmission and network activities. At the start of the nineties a number of pioneering studies showed that glutamate applications in cell cultures and brain slice preparations evoked Ca^2+^ elevations and propagating Ca^2+^ waves in astrocytes (Dani et al., 1992). Subsequent observations revealed that Ca^2+^ elevations in astrocytes triggered glutamate release that evoked AMPA and NMDA receptor-mediated Ca^2+^ increases in nearby neurons (Nedergaard, 1994; Parpura et al., 1994; Pasti et al., 1997). The indication of an astrocyte-to-neuron signaling represented a turning point in astrocyte research and paved the way to studies in brain slice preparations and *in vivo* which essentially confirmed the existence of a complex bidirectional signaling between neurons and astrocytes (Zonta and Carmignoto, 2002). We now know that astrocytes express a large variety of receptors that can be activated by various neurotransmitters, such as glutamate (Porter and McCarthy, 1996; Pasti et al., 1997), GABA (Kang et al., 1998), noradrenaline (Duffy and MacVicar, 1995; Kulik et al., 1999), acetylcholine (Shelton and McCarthy, 2000; Araque et al., 2002), ATP, and also by cannabinoids released by neuronal dendrites (Navarrete and Araque, 2008, 2010). These molecules evoke Ca^2+^ elevations in astrocytes that, in turn, release other transmitters including glutamate, d-serine, and ATP. By means of these transmitters, termed gliotransmitters, astrocytes affect neuronal excitability, and modulate synaptic transmission. Beside these gliotransmitters astrocytes can release cytokines such as IL-1β and TNFα, as well as other molecules such as TGFβ, GDNF, BDNF, nitric oxide, and neurosteroids that can also affect neuronal functions. The ability of astrocytes to listen and talk to synapses by exerting both excitatory and inhibitory actions on neurons (Pasti et al., 1997; Araque et al., 1999; Brockhaus and Deitmer, 2002; Zhang et al., 2003; Pascual et al., 2005; Panatier et al., 2006, 2011; Serrano et al., 2006; Jourdain et al., 2007; Perea and Araque, 2007; Di Castro et al., 2011; Shigetomi et al., 2011; Min and Nevian, 2012; Navarrete et al., 2012) is revolutionizing our original view that information processing in the brain is exclusively based on billions of neurons interacting dynamically in the neuronal network. On the other hand, clues for a distinct contribution of astrocytes in the highest functions of the mammalian brain during phylogeny are the increasing complexity and size of protoplasmic astrocytes with respect to the unchanged features of cortical neurons, and the relative expansion in the number of astrocytes with respect to that of neurons (Oberheim et al., 2006, 2009). All in all, the astrocyte is now proposed to represent an intrinsic element of the neuronal circuit that composes a tripartite synapse with the pre- and the post-synaptic neuronal membrane (Araque et al., 1999; Carmignoto, 2000; Haydon and Carmignoto, 2006; Halassa et al., 2009; Perea et al., 2009).

The discovery that astrocytes modulate neuronal communication and are involved in the control of brain network activities suggest that astrocytes have the potential to be involved in the development of a number of brain disorders, from acute brain injury or infection to chronic diseases including Alzheimer’s diseases, Parkinson disease, amyotrophic lateral sclerosis, and epilepsy (Maragakis and Rothstein, 2006; Seifert et al., 2006; Blackburn et al., 2009). The aforementioned observations on Ca^2+^-dependent release of glutamate in astrocytes providing direct excitation to neighboring neurons is of particular interest in the context of epileptogenesis. Accordingly, alterations in this astrocyte-derived excitatory pathway in concert with impaired astrocytic glutamate and K^+^ uptake may result in a hyperexcitable neuron-astrocyte network which favors neuronal synchronization and ultimately predisposes neurons to seizures. In this review we present an overview of the “historical” observations that suggested a role of astrocytes in the epileptic brain and we also critically discuss the most recent results that hint at a distinct contribution of astrocytes in the generation of focal epileptiform activities.

## Astrocytes in the Epileptic Brain

It has been known for a long time that early in the development of many brain disorders, astrocytes exhibit significant morphological and functional changes (Hamby and Sofroniew, 2010). This condition, termed reactive gliosis, comprises increased number, altered morphology with hypertrophic soma and processes, spatial overlapping and different functional changes of astrocytes. Gliosis is frequent in the human epileptic brain and seizures have been shown to frequently initiate within or very near gliotic brain tissues (McKhann et al., 2000). Gliosis is frequently observed in temporal lobe epilepsies (TLE), a severe form of partial epilepsy with focal onset in temporal structures such as hippocampus, entorhinal cortex, and amygdala. Surgical ablation of the epileptogenic region may be the only treatment available in the most severe forms of TLE that are resistant to all the currently available AEDs. Studies from these tissues, as well as from post-mortem tissues or from animal models, have shown relevant alterations of neuronal and astrocytic morphology. It is important to point out that it is still unclear whether an altered glial morphology can contribute to the generation of the disorder or be a consequence of an established damage of the brain tissue. This especially holds for human tissues from patients that may be suffering from the pathology since many years, rather than for animal models in which investigations can be more easily performed at different steps of the disease.

The most frequent and striking alteration in TLE is hippocampal sclerosis which is characterized by a massive gliosis, neuronal loss, synaptic, and microvascular reorganization. Gliosis, that can be revealed by increased expression of glial fibrillary acidic protein (GFAP, an intermediate filament protein), has been described also in other forms of epilepsies like post-traumatic and infection-induced epilepsy (Seifert et al., 2006). In animal models, including the pilocarpine- and the kainate-induced epilepsy, gliosis is also early observed during the period that precedes recurrent seizure onset, suggesting a causative role of astrocytes in epileptogenesis (Represa et al., 1995; Belluardo et al., 1996; Bouilleret et al., 1999; Borges et al., 2003; Rizzi et al., 2003).

While we have only a limited knowledge of the functional changes occurring in reactive astrocytes, we can reasonably advance the hypothesis that an astrocyte dysfunction contributes or even plays a central role in epileptogenesis. Notably, although astrocyte abnormalities have been, in general, linked to proconvulsant actions, astrocyte signals can also exert an anticonvulsants action in a context-dependent manner. In the following sections we will examine to what extent an impairment of a distinct astrocytic function may contribute to modulate brain network excitability ultimately favoring, or opposing, the generation and spread of seizure-like discharges. In view of the recognized role played by gliotransmission in various forms of synaptic plasticity (Serrano et al., 2006; Florian et al., 2011; Panatier et al., 2011; Min and Nevian, 2012; Navarrete et al., 2012), it is worth underlying that the cognitive deficits observed in a number of neurological disorders, including epilepsy, may be due, at least in part, to an impairment of the reciprocal signaling between neurons and astrocytes.

## Potassium Homeostasis

A correct K^+^ homeostasis is a crucial factor in the control of neuronal excitability. Because even small elevations in the extracellular K^+^ concentrations ([K^+^]_o_) can significantly increase neuronal network activity, it was hypothesized that [K^+^]_o_ is a causal factor for epileptiform activity. Indeed, perfusion of brain slices with high K^+^ is effectively used to induce epileptic-like discharges in neurons. Moreover, a bulk of experimental studies performed in different *in vitro* and *in vivo* models of epilepsy showed that seizure activity itself can lead to a significant extracellular K^+^ increase up to 10–12 mM (Pedley et al., 1976; Heinemann et al., 1977, 1986; Lopantsev et al., 2009). A number of early studies also revealed a high and rapid permeability of glial cells to K^+^ ions, even in response to small neuronal activities (Kuffler et al., 1966; Orkand et al., 1966). In these initial studies the so-called “spatial potassium buffering hypothesis” was advanced. According to this hypothesis, astrocytes either singularly or connected through gap-junctions, were proposed to redistribute local [K^+^]_o_ increases either to neighboring regions or directly into the blood vessels (Orkand et al., 1966; Kofuji and Newman, 2004). Accordingly, astrocytes are thought to play a predominant role in the clearance of the K^+^ ions which accumulate in the extracellular space during the intense neuronal firing that characterizes epileptic discharges. Inward rectifying K^+^ channels (Kir) are the main responsible for the high astrocytic K^+^ permeability. Of the 16 distinct Kir channel subunits which have been identified, type 4.1 (Kir4.1) and, to a lesser extent, type 2.3 (Kir2.3) are the most intensively studied (Neusch et al., 2006; Olsen and Sontheimer, 2008). Notably, astrocytes from different brain regions abundantly express Kir4.1 channels (Takumi et al., 1995; Ishii et al., 1997; Poopalasundaram et al., 2000; Higashi et al., 2001; Li et al., 2001). Unlike other K^+^ channels that allow large K^+^ efflux from depolarized cells, such as those that underline membrane repolarization during action potential firing in neurons, Kir channels have the peculiarity to mediate small K^+^ efflux from depolarized cells and large influx at hyperpolarized potentials. Most importantly, due to a high open probability at resting potential (Ransom and Sontheimer, 1995) and a channel conductance that is proportional to [K^+^]_o_ (Sakmann and Trube, 1984; Newman, 1993), Kir4.1 channels can be effectively activated by large [K^+^]_o_ increases and mediate a large K^+^ influx.

Several studies showed altered Kir4.1 expression and activity in epilepsy, both in humans and experimental animal models, as well as in other disorders including brain injury. A reduction of astrocytic Kir4.1 channels expression was observed in several studies on tissues from TLE patients (Bordey and Sontheimer, 1998; Hinterkeuser et al., 2000; Kivi et al., 2000; Schroder et al., 2000). Kir currents recorded in astrocytes from human sclerotic hippocampi were also reduced (Heinemann et al., 2000; Hinterkeuser et al., 2000). It is, however, unclear whether these changes are a reaction of the tissue to the epileptiform activities or whether they are directly involved in the genesis of the disease. The use of transgenic animals helped to address this issue and results obtained confirmed the importance of Kir channels during epileptogenesis. In particular, Kir4.1 knock-out mice exhibited an impaired K^+^ homeostasis in the retina (Kofuji et al., 2000) and the brain stem (Neusch et al., 2006), and showed stress-induced seizures (Djukic et al., 2007). In a recent *in vivo* study in Kir4.1 conditional knock-out mice, the central role of Kir4.1 channels in the [K^+^]_o_ regulation in the hippocampal region was also reported (Chever et al., 2010). Another compelling, although indirect, evidence for the Kir involvement in the generation of epileptiform activities is the finding that mutations or polymorphisms of KCNJ10, the gene encoding Kir4.1 subunits, have been associated with increased seizure susceptibility and epileptic phenotypes in mice (Ferraro et al., 2004; Inyushin et al., 2010). Humans KCNJ10 mutations are also associated to seizure susceptibility, idiopathic generalized epilepsy (Buono et al., 2004; Lenzen et al., 2005) or to complex syndromes that encompass epilepsy (Bockenhauer et al., 2009; Scholl et al., 2009; Reichold et al., 2010). Notably, a reduced Kir4.1-mediated K^+^ buffering, due to conditional knock-out of Kir4.1 channels or polymorphism of KCNJ10, has been linked to a reduced glutamate clearance (Djukic et al., 2007; Kucheryavykh et al., 2007; Inyushin et al., 2010) that further contributes to increase network excitability. Altogether, these observations support the view that either a reduced Kir channel expression or a functional impairment of these channels due to neuronal injury or gene polymorphisms can increase network activity leading to a higher susceptibility to proconvulsant stimuli or to generation of a full epileptic activity. As crucial regulators of [K^+^] and network excitability, astrocytic Kir channels represent potential targets for a novel therapeutic approach for epilepsies.

## Water Homeostasis

Water homeostasis is another aspect of great importance in brain pathophysiology in which astrocytes play a pivotal role. Different conditions such as brain ischemia, injury, or hepatic encephalopathy can result in cerebral edema that is mainly due to water accumulation in astrocytes. Astrocyte swelling leads to a reduction of the extracellular space that enhances network excitability and favors epileptiform discharges (Dudek et al., 1990; Andrew, 1991; Roper et al., 1992; Chebabo et al., 1995; Pan and Stringer, 1996), possibly due to an increase of ephaptic interactions between neurons. Astrocyte swelling can also result in an increased release of gliotransmitters, such as glutamate, via volume-sensitive organic anion channels (VSOACs), that further enhances network excitability. Water influx in astrocytes is meditated by the water channel aquaporin 4 (AQP4) that is, indeed, highly expressed in these cells (reviewed by Badaut et al., 2002, 2007) with a prevalent localization in perivascular endfeet (Nielsen et al., 1997; Higashi et al., 2001; Nagelhus et al., 2004). The peculiar anchoring of AQP4 on perivascular endfeet seems to rely on α-syntrophin, a dystrophin complex protein (reviewed by Amiry-Moghaddam et al., 2004) that is important to link intracellular water content to blood circulation. Interestingly, AQP4 channels colocalize with Kir4.1 (Nagelhus et al., 2004) and AQP4 deficient or knock-out mice show a slowed K^+^ influx. These latter mice also show prolonged seizures (Amiry-Moghaddam et al., 2003; Binder and Steinhauser, 2006). These and other studies support the hypothesis that AQP4 and Kir4.1 channels cooperate in the regulation of water and K^+^ homeostasis in brain tissue. Like Kir4.1 channels, AQP4 are also altered in the sclerotic human hippocampi with an overall increase of expression (Lee et al., 2004), but a redistribution from the perivascular site to the perisynaptic one (Lee et al., 2004). Similar results are also observed in epileptic tissue with focal cortical dysplasia (FCD), a cortical malformation that frequently generates epileptic foci (Medici et al., 2011). In another recent study, a decreased expression of AQP4 has been observed in the kainic acid model of epileptogenesis in wild-type mice together with an increase of seizures occurrence in AQP4−/− mice (Lee et al., 2012). An altered distribution of AQP4 may thus reduce the transfer of water from astrocytes into blood vessels. This would lead to astrocyte swelling, reduced clearance of both extracellular K^+^ and glutamate as well as an increase in glutamate release, also through volume-sensitive anion channels, triggering a deleterious loop that can further facilitate cell swelling. A recent work has shown a critical role of AQP4 also for astrocytic Ca^2+^ signaling (Thrane et al., 2011). In particular, the authors showed that hypo-osmotic stress triggers Ca^2+^ increases in astrocytes and this response depends on AQP4 and, partially, on P2Y receptors. Consistent with an important role of AQ-4 in astrocytic Ca^2+^ signaling and astrocyte-neuron interactions, AQ-4 knock-out mice exhibited a cognitive defect and a reduction of a distinct form of hippocampal LTP (Skucas et al., 2011). Altogether, the currently available data suggest a strong involvement of astrocytic water channels in epileptogenesis (for review see Binder et al., 2012).

## Glutamate and GABA

According to a classical paradigm epileptiform activities may arise from a brain network that becomes hyperexcitable by an imbalance in glutamatergic and inhibitory GABAergic transmission (Prince and Wilder, 1967; Bradford, 1995). This simple view has been recently revisited taking into account that GABAergic transmission may favor epileptic activity by synchronizing large neuronal populations and even depolarize, at least under certain conditions, the neuronal membrane thereby enhancing neuronal excitability (Cossart et al., 2005; Fritschy, 2008; Avoli and de Curtis, 2011). Although the ultimate action of GABAergic transmission may be context-dependent, seizure threshold is strongly affected by the dynamics of both glutamate and GABA signals. The tonic extracellular level of these neurotransmitters can also contribute to control network excitability. As regards glutamate, the large amount of this neurotransmitter, released into the extracellular space by the activity of both glutamatergic synapses and astrocytes, is internalized by an efficient reuptake system that includes five different excitatory amino acid transporters, EAAT1-5. EAAT1 and EAAT2 are selectively expressed in astrocytes (GLAST and GLT-1 in rodents, respectively) and they represent the main regulators of extracellular glutamate levels in the brain (Rothstein et al., 1994; Tanaka et al., 1997). An additional factor that contributes to set the extracellular glutamate concentration in the brain is the glutamate metabolism. According to the glutamate-glutamine cycle, glutamate is mainly synthesized in astrocytes starting from the glucose internalized by astrocyte endfeet in contact with cerebral blood arterioles. Glucose is then converted to glutamate (via α-ketoglutarate) and successively to glutamine by the specific astrocytic enzyme glutamine synthetase (GS). Glutamine released from astrocytes is captured by glutamatergic neurons through specific transporters and finally deaminated to glutamate by phosphate activated glutaminase. In astrocytes the extracellular glutamate is internalized and converted again into glutamine that is then transferred to neurons to start a new cycle.

High extracellular glutamate levels have been reported in human epileptic tissues (During and Spencer, 1993; Glass and Dragunow, 1995; Cavus et al., 2005). These observations lead to the hypothesis that an increased glutamatergic transmission may be an early event that contributes to the generation of distinct types of epilepsy. Controversial results have been, however, reported in studies investigating the EAAT levels from tissues of TLE patients (reviewed by Eid et al., 2008b; Seifert et al., 2010). While some studies revealed a down regulation of astrocytic glutamate transporters (Mathern et al., 1999; Proper et al., 2002; Sarac et al., 2009), others failed to observe similar alterations (Tessler et al., 1999; Eid et al., 2004). The potential of astrocytic EEAT transporters to participate in seizure generation was, however, demonstrated in a transgenic mouse line in which a deletion of EATT2 in astrocytes resulted in epileptiform activities (Tanaka et al., 1997). In addition, in an animal model of focal epilepsy induced by a blood brain barrier (BBB) disruption that mimics post-traumatic epilepsy, a reduced glutamate and K^+^ clearance capacity was reported (David et al., 2009; see also below).

A more general consensus regards the loss of GS in the hippocampus from TLE patients that showed elevated extracellular glutamate levels (Eid et al., 2004; van der Hel et al., 2005). Reduced GS levels and activity are thought to increase extracellular glutamate levels because the intracellular conversion of glutamate to glutamine favors the uptake of glutamate from extracellular space (Otis and Jahr, 1998). It was recently shown that inhibition of GS by methionine sulfoximine induced recurrent seizures in animal models (Eid et al., 2008a; Wang et al., 2009). Notably, astrocytic glutamine is not only the main source of glutamate in pyramidal neurons, but it also produces GABA in GABAergic interneurons. Among the different interneurons, the fast-spiking subtype demands a very efficient GABA recycling system due to the very high frequency of action potential firing in these cells. Indeed, the inhibition of astrocytic GS in experimental animal models leads to reduced inhibitory synaptic currents and GABA release without similar changes in the glutamatergic pathway (Liang et al., 2006; Kam and Nicoll, 2007; Ortinski et al., 2010). Consistent with this view, reduced levels and activity of GS in the epileptic brain were observed to favor seizure generation by increasing extracellular glutamate and decreasing GABA release at presynaptic terminals. Interestingly, in a recent study in different genetic models of absence seizures – a form of thalamic epilepsy with excessive GABA levels – a reduced GABA uptake in astrocytes was found to be a causal factor for this epileptic phenotype, pointing out at an astrocytic dysfunction as a main cause also for this type of epilepsy (Cope et al., 2009). Although some controversial aspects need to be addressed, results from these studies support the hypothesis that altered glutamate/GABA metabolism and uptake in astrocytes can play an important role in the epileptic brain.

While the interactions between GABAergic interneurons and astrocytes have been poorly addressed in the context of epilepsy, it is noteworthy that astrocytes are also a source of neurosteroids, a class of potent endogenous modulators of GABA_A_ receptors with sedative, anxiolytic, and anticonvulsive properties (Lambert et al., 2003; Reddy, 2010). Neurosteroids such as allopregnanolone (3α-hydroxy-5α-pregnane-20-one) and allotetrahydrodeoxycorticosterone (THDOC; 3α,21-dihydroxy-5α-pregnan-20-one), can either originate in peripheral organs or be synthesized *de novo* in the brain by both neurons and astrocytes (Mensah-Nyagan et al., 1999; Agis-Balboa et al., 2006). Interestingly, the cytochrome P450 cholesterol side-chain cleavage enzyme (P450scc) that catalyzes the initial step of neurosteroid biosynthesis in the brain was found to be increased in hippocampal astrocytes during the latent period after pilocarpine-induced status epilepticus (SE) in rats (Biagini et al., 2006, 2009). In these studies longer SE was correlated to higher P450scc expression in astrocytes and longer latent period, while blockade of neurosteroid synthesis accelerated seizure onset, suggesting that activated astrocytes may temporarily prevent spontaneous seizure onset by producing neurosteroids. These data raise the hypothesis of an anticonvulsant action of astrocytes and press for additional studies aimed to clarify their role in the production of neurosteroids and of other modulators of neuronal excitability.

## Gliotransmission: Glutamate Release

The excessive brain network excitability and the hypersynchronous discharges that are observed in epileptic disorders are believed to represent abnormalities intrinsic to neurons. In the generation of epileptiform activity non-neuronal mechanisms may, however, also contribute (Konnerth et al., 1986; Dudek et al., 1998; Jefferys, 2003). Astrocytes can represent, indeed, an extrasynaptic source of glutamate that triggers synchronized activities in groups of neurons from different brain regions (Angulo et al., 2004; Fellin et al., 2004, 2007). Paired patch-clamp recording experiments from two pyramidal neurons revealed that the release of glutamate from astrocytes – in response to Ca^2+^ elevations evoked by different agents or direct mechanical stimulation – induced in both neurons *N*-methyl-d-aspartic acid (NMDA) receptor-mediated slow inward currents (SICs) that could occur with a high level of synchrony (Angulo et al., 2004; Fellin et al., 2004). Ca^2+^ imaging experiments also revealed that astrocyte Ca^2+^ elevations could be followed by episodes of simultaneous Ca^2+^ elevations in small groups of adjacent neurons, a response that we termed a “domain response” (Fellin et al., 2004). It resulted that stimuli commonly used to activate a Ca^2+^ signal in astrocytes, such as agonists of type I metabotropic glutamate receptors (mGluRs), purinergic, and prostaglandin E2 receptors, triggered in the presence of TTX glutamate release from astrocytes and synchronous activity in neurons (Fellin et al., 2004, 2006b). Given that the Ca^2+^ dependent release of glutamate from astrocytes could promote local synchronous activities in hippocampal neurons, it was reasonable to propose that an excessive activation of Ca^2+^ elevations in astrocytes may result in a massive glutamate release and represent a relevant non-neuronal mechanism of hypersynchronous discharges in neurons. In support of this view, studies performed both on brain slices and *in vivo* showed that during epileptiform activity, the frequency of Ca^2+^ oscillations in astrocytes is significantly increased (Tian et al., 2005; Fellin et al., 2006a), while it is reduced by anticonvulsant drugs (Tian et al., 2005). Moreover, mGluR expression in hippocampal astrocytes from animal models of temporal lobe epilepsy was also found to be increased (Aronica et al., 2000; Ulas et al., 2000). Furthermore, glutamate released from rodent astrocytes during SE contributes to neuronal death (Ding et al., 2007). This process could be averted by inhibiting glia-neuron signaling and by applying mGluR5 and NR2B NMDA receptor antagonists which suppress astrocytic Ca^2+^ increase and block extrasynaptic NMDA receptors activated by glia-derived glutamate (Ding et al., 2007). These observations support the hypothesis that an increase excitability in the neuron–astrocyte network favors neuronal synchronization and ultimately predisposes neurons to seizures.

In five different experimental models of chemically induced epilepsy, 70–90% of paroxysmal depolarizing shifts (PDSs), i.e., interictal spikes, were found to be insensitive to tetrodotoxin applications (Kang et al., 2005; Tian et al., 2005). Given that astrocytic glutamate plays a prominent role in the generation of such a large fraction of PDSs, SICs coincide, according to these authors, with PDSs and seizure activity may have an astrocytic basis (Tian et al., 2005). This conclusion, however, fueled a controversial debate on the role of astrocytes in epileptogenesis since it contradicts a large number of previous studies showing that TTX efficiently blocks PDS (D’Ambrosio, 2006; Seifert et al., 2006; Wetherington et al., 2008). The conclusion by Tian et al., was also disputed by subsequent studies which further confirmed the sensitivity of PDS to TTX (Fellin and Haydon, 2005; Gomez-Gonzalo et al., 2010). On the other hand, it is rather unlikely that astrocyte-mediated SICs in pyramidal neurons represent PDSs. SICs have been demonstrated to be mediated exclusively by NMDA receptors (Angulo et al., 2004; Fellin et al., 2004; Perea and Araque, 2005), while PDSs were reported to be, at least in large part, insensitive to D-AP5. As reviewed by Wetherington et al. (2008) a huge amount of data strongly support the conclusion that SICs and PDSs, in spite of a similar time course, are distinct events and derive from a different cellular source.

**Figure 1:**
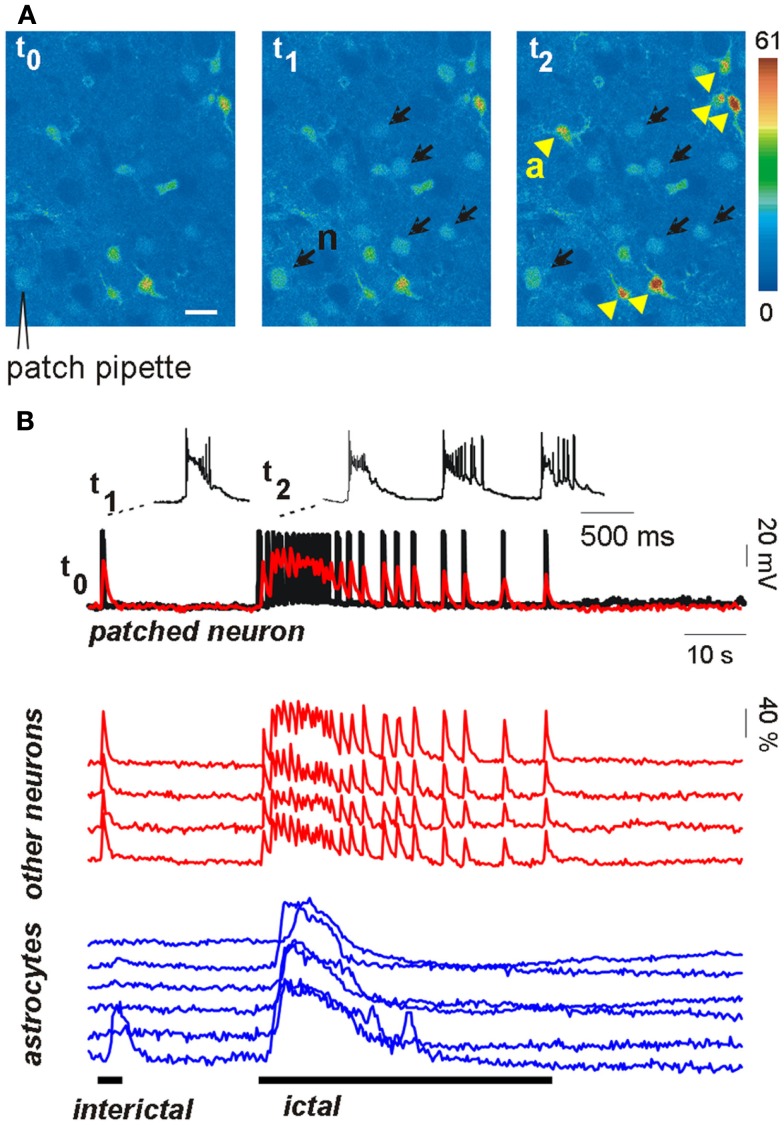
**Astrocytes are activated during focal seizure propagation in cortical slices**. **(A)** Image sequence of the fluorescence signal change in layer V–VI neurons (n, black arrows) and astrocytes (a, yellow arrowheads) from the entorhinal cortex of a young rat after slice loading with the Ca^2+^ dye Oregon-Green BAPTA1-AM during a propagating ictal discharge. **(B)** Simultaneous patch-clamp recording from one neuron [black trace; the patch-pipette is indicated in **(A)**] and Δ*F*/*F*_0_ traces of the Ca^2+^ signal in the same neuron (superimposed red trace) and other neighboring neurons (red traces) and astrocytes (blue traces). The Ca^2+^ signal from all neurons precisely matched the electrical activity of the patched neuron during both the interictal event (single event at trace onset) and the subsequent ictal discharge. Astrocytes were strongly activated during the ictal discharge while they were poorly activated by the interictal discharge (adapted from Gomez-Gonzalo et al., 2010).

The results reported above do not rule out the possibility, however, that astrocytic glutamate is an important factor in epileptiform activities (Fellin et al., 2006a). A recent study from our group helped to clarify the effective weight of the astrocyte contribution to the generation of epileptic discharges (Gomez-Gonzalo et al., 2010). We found that astrocytes could contribute to seizure-like ictal discharge, but not to interictal discharge arising spontaneously in our picrotoxin/low Mg^2+^ cortical slice model. Indeed, astrocyte Ca^2+^ elevations were observed to regularly accompany the generation of the ictal discharge, but these events were observed only rarely in a few astrocytes during the interictal discharges (Figure 1). To further define the role of astrocytes in the generation of epileptiform activities, we also used additional models of epileptic seizures, including a new model of stimulus-evoked seizures (Losi et al., 2010). In the new model, an episode of hyperactivity that is induced in a small group of neurons by local NMDA applications in the presence of the proconvulsant 4-amino pyridine (4-AP), generates an ictal, seizure-like discharge that from this epileptogenic site propagates to adjacent neuronal populations. The unique advantage of the new model is that we know in advance when and where a focal seizure will occur, giving us the opportunity to investigate the earlier cellular events that develop at the epileptogenic site during the preictal, transition phase, and predispose neurons to seizures. We found that neuronal hyperactivities at these restricted brain sites are accompanied by an early Ca^2+^ elevation in a large number of astrocytes (see Figure 1) that contributes to drive neurons toward the threshold of seizure discharge generation (Gomez-Gonzalo et al., 2010). Indeed, the onset of Ca^2+^ elevations in astrocytes from the epileptogenic site occurred a few seconds before the initial development of focal, seizure-like discharges. Most importantly, we were able to show that the early astrocyte activation was not a mere consequence of the episode of high neuronal activity triggered by NMDA and it rather represents a crucial step in the generation of the ictal discharge. After the Ca^2+^ chelator BAPTA was introduced into the astrocyte syncytium by patching individual astrocytes with a BAPTA-containing pipette, the Ca^2+^ elevations in astrocytes were strongly reduced and the NMDA stimulation that was normally successful failed to evoke an ictal discharge. According to results obtained from different control experiments, the effect of BAPTA on ictal discharge generation was specifically linked to the inhibition of astrocyte Ca^2+^ signals, and, possibly, to the inhibition of gliotransmitter release, such as glutamate and d-serine, that can favor synchronous neuronal activities.

These results suggest that when an episode of hyperactivity in a group of neurons consistently engages nearby astrocytes, a feedback signal from these latter cells enlarges the population of neurons recruited into a coherent activity. If this signal operates on a brain network prone to seizures, neurons are driven toward the ictal discharge threshold. It is worthwhile to emphasize here that when the astrocyte contribution was reduced, for example following BAPTA intracellular applications, the ictal discharge was inhibited, but it could be recovered by applying a higher NMDA stimulation (Gomez-Gonzalo et al., 2010). By activating directly a larger number of neurons, this higher stimulus evokes a level of correlated activity that is sufficient for seizure-like discharge generation, bypassing the astrocyte contribution in the recruitment process. Thus, astrocytes are not an absolute requirement, but they can provide an important and even, under certain circumstances, crucial contribution to ID generation.

## Gliotransmission: ATP Release

Another gliotransmitter that plays a relevant role in the control of epileptic activity is ATP. The effect of ATP can be even more complex than that of glutamate and exert in the neuronal network both pro and anticonvulsant actions. ATP is released from astrocytes either through a Ca^2+^-dependent (Coco et al., 2003; Pascual et al., 2005) or a Ca^2+^-independent mechanism, the latter involving connexin and pannexin hemichannels (for a review, see Burnstock et al., 2011). Astrocytic ATP and its main metabolite adenosine (Ado) exert a plethora of actions on astrocytes, neurons, microglia, oligodendrocytes, and blood vessels through different purinergic receptors. ATP is responsible for the slow Ca^2+^ waves that propagate across the astrocytic syncytium by acting on P2Y receptors (Cotrina et al., 1998; Koizumi, 2010).

While ATP can directly excite neurons through P2 receptors with pro-epileptic effects (for a review, see Kumaria et al., 2008), it might also have an anticonvulsant action due to activation of P2Y receptors on inhibitory interneurons (see below) or for the effects of its metabolite Ado. This strongly inhibits excitatory neurotransmitter release through activation of presynaptic A1 receptors and is considered, indeed, a potent endogenous anticonvulsant (for reviews see Li et al., 2007; Boison, 2008, 2010). Ado derived from astrocytic ATP is also at the basis of some distinct forms of synaptic plasticity such as the heterosynaptic depression in the hippocampus (Zhang et al., 2003, p. 6691; Pascual et al., 2005, p. 7468; Serrano et al., 2006, p. 8330). In light of these findings astrocytic ATP may initially favor (via P2 receptors) and then inhibit epileptic activity after its conversion to Ado (via A1 receptors). Noteworthy is also that astrocytes are major regulators of extracellular Ado levels since these cells are not only its major source but they also predominantly express adenosine kinase (ADK), an enzyme that efficiently degrades Ado (Gouder et al., 2004; Boison, 2005). In the epileptic brain, including the hippocampus from patients with temporal lobe epilepsy, ADK is increased resulting in decreased extracellular Ado concentrations that favor network excitability (Gouder et al., 2004; Aronica et al., 2011). Notably, cell grafts releasing Ado and genetic reduction of ADK were shown to prevent seizures and reduce epileptogenesis in animal models of epilepsy (Huber et al., 2001; Li et al., 2007, 2008). These observations suggest that ADK can be considered not only a diagnostic marker of epilepsies, but also a potential target for the development of a new therapy that could prevent epileptic seizures (Boison, 2008; Theofilas et al., 2011).

Interestingly, a recent study showed that a local Ca^2+^ decrease in the extracellular space initiates a Ca^2+^ wave in astrocytes specifically mediated by astrocytic ATP release through connexin 43 hemichannels (Torres et al., 2012). This astrocytic ATP enhances inhibitory transmission by acting on the P2Y1 receptors expressed in a subset of hippocampal interneurons (Torres et al., 2012). Given that during epileptic activity the extracellular Ca^2+^ is markedly reduced, the consequent release of astrocytic ATP could potentiate inhibitory transmission thereby working as an anticonvulsant feedback mechanism that opposes seizure propagation.

All in all, it can be predicted that ATP gliotransmission may soon be recognized as target for developing new antiepileptic therapies.

## Inflammation

A large variety of brain insults including stroke, acute infections, trauma, febrile seizures, and cancer are associated with a high risk of developing epilepsy and all these conditions are known to trigger a rapid inflammatory reaction in the brain tissue. The activation of inflammatory pathways appears, indeed, to play an important role in the etiopathogenesis of several forms of epilepsy (Turrin and Rivest, 2004; Vezzani and Granata, 2005; Vezzani et al., 2008; Fabene et al., 2010). Among the inflammatory agents that are highly expressed in the epileptogenic tissue are the pro-inflammatory cytokines IL-1β and TNFα. Main sources of these cytokines are microglia and astrocytes. The mechanism at the basis of the IL-1β proconvulsant action is not completely understood. Recent works suggest that IL-1β acts on neuronal IL-1β receptor 1 (IL-1R1), the IL-1R accessory protein, myeloid differentiation primary response protein complex (MyD88) and Src family kinases to ultimately enhance NMDA-mediated Ca^2+^ influx by increasing the phosphorylation of the NR2B subunit (Viviani et al., 2003; Balosso et al., 2008). Other mechanisms are, however, possible. In a recent study in brain slices IL-1β was reported to induce hyperexcitability independently of NMDA receptors (Rossi et al., 2012). The authors report an increased occurrence of AMPA receptor-mediated spontaneous excitatory synaptic currents due to the action of IL-1β on transient receptor potential vanilloid 1 (TRPV1) channels.

It is worth underlying that IL-1β and TNFα were also reported to act on astrocytes, significantly reducing glutamate uptake and increasing glutamate release in these cells (Ye and Sontheimer, 1996; Hu et al., 2000; Bezzi et al., 2001; Santello et al., 2011) raising the possibility that the proconvulsant action of these cytokines can be linked, at least in part, to a direct effect on astrocytes. In a series of recent work it was also shown that in neocortex the disruption of the BBB, an event frequently associated with neuroinflammation, leads to a rapid astrocyte activation with increased GFAP expression. This is due to albumin diffusion which is selectively captured by astrocytes through a mechanism involving TGF-β receptors. Indeed, an increased BBB permeability is proposed to contribute to the generation of an epileptogenic focus (Seiffert et al., 2004; Ivens et al., 2007; Tomkins et al., 2007; Friedman et al., 2009). Importantly, the rapid gliosis that follows a disruption of the BBB was associated with a reduced expression of Kir channels, gap-junctional proteins, and glutamate transporters in astrocytes that lead to an increased network excitability by reducing K^+^ and glutamate uptake (David et al., 2009).

The high-mobility group box-1 (HMGB1) is an additional inflammatory agent (Müller et al., 2004; Pedrazzi et al., 2007; Sims et al., 2010) recently revealed to be involved in seizure generation (Maroso et al., 2010). HMGB1 is produced by both neurons and glia and its action appears to be mediated by the toll-like receptor 4 (TLR4), a receptor that recognizes several pathogenic microbial molecules (Maroso et al., 2010).

Although many molecular aspects remain to be elucidated, these studies provide significant support to the hypothesis that astrocytes are directly involved in the inflammatory reaction that favor the development of epilepsy.

## Conclusions and Perspectives

**Figure 2:**
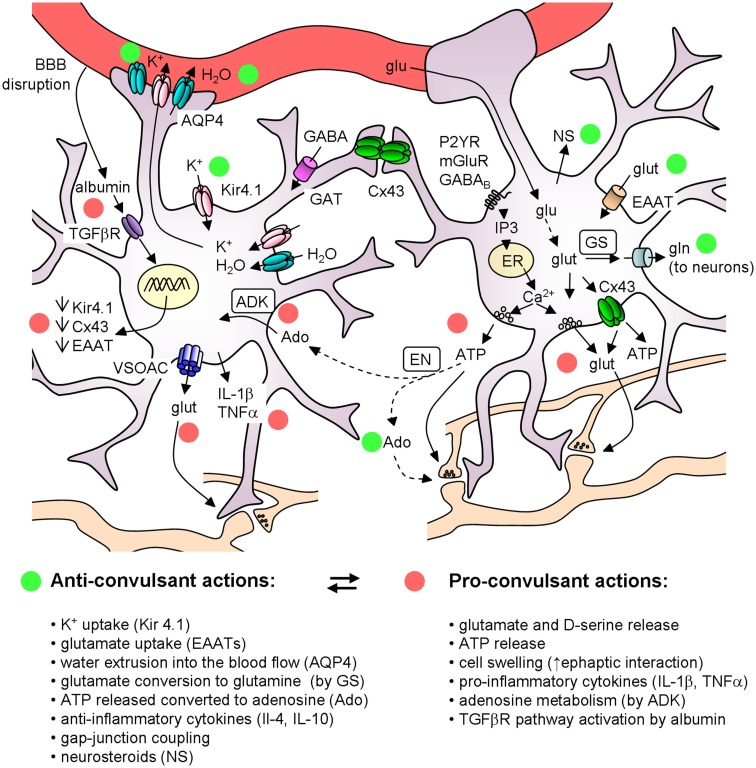
**Schematic of the main astrocyte actions that can affect epileptiform activities**. The colored circles mark the anticonvulsant (green) and the proconvulsant (red) actions of astrocytes. Note that (i) ATP can have *per se* a proconvulsant action, but after its conversion to Ado it is anticonvulsant; (ii) astrocytic cytokines can have either pro- or anti-inflammatory actions. ADK, adenosine kinase; Ado, adenosine; AQP4, aquaporin 4; BBB, blood brain barrier; Cx43, connexin 43; EAAT, excitatory amino acid transporters; EN, ectonucleotidase; ER, endoplasmatic reticulum; GAT, GABA transporters; glu, glucose; glut, glutamate; gln, glutamine; GS, glutamine syntethase; NS, neurosteroids; VSOACs, volume-sensitive organic anion channels.

Astrocytes are key players in the regulation of brain tissue homeostasis and neuronal excitability. A large body of evidence showed the impairment of several astrocytic functions in the epileptic brain, such as the control of water and ionic equilibrium, neurotransmitter concentrations, cerebral blood flow, and the ability to release modulators and cytokines. Although a dysregulation of a number of astrocyte-specific functions has been identified and linked to epilepsies, the precise role of gliotransmission in initiation and propagation of seizures is unclear and many key questions remain unanswered. Among these questions it is relevant to clarify if and under which conditions the different gliotransmitters exert a pro or an anticonvulsant action (see Figure 2). Very likely, the signaling of astrocytes to principal neurons and distinct interneurons through glutamate release may be context-dependent and needs selective tools to be elucidated. ATP-mediated gliotransmission is another promising field for future experiments given its role in mediating Ca^2+^ waves in the astrocytic syncytium and its wide effects on neuronal functions. Accordingly, a clarification of ATP release mechanisms could potentially allow the development of selective tools to modulate gliotransmission and network excitability. As to epileptogenesis, an important issue is whether astrocytes favor the generation of epileptic events or may, instead, oppose it by releasing, for example, molecules like neurosteroids and trophic factors. To address these questions a great help is provided by new experimental approaches that use molecular genetic tools to selectively affect specific signaling pathways in astrocytes as well as by the application of Ca^2+^ imaging techniques in *in vivo* animal models of epilepsy.

Clarification of the astrocyte role in brain disorders represents one of the most difficult, but also one of the most fascinating challenges in neurobiological research in the years to come.

## Conflict of Interest Statement

The authors declare that the research was conducted in the absence of any commercial or financial relationships that could be construed as a potential conflict of interest.
